# Constitutive activation of MET signaling impairs myogenic differentiation of rhabdomyosarcoma and promotes its development and progression

**DOI:** 10.18632/oncotarget.5145

**Published:** 2015-09-08

**Authors:** Klaudia Skrzypek, Anna Kusienicka, Barbara Szewczyk, Tomasz Adamus, Ewa Lukasiewicz, Katarzyna Miekus, Marcin Majka

**Affiliations:** ^1^ Department of Transplantation, Polish-American Institute of Pediatrics, Jagiellonian University Medical College, 30-663 Krakow, Poland; ^2^ Department of General Biochemistry, Faculty of Biochemistry, Biophysics and Biotechnology, Jagiellonian University, 30-387 Krakow, Poland

**Keywords:** MET, rhabdomyosarcoma (RMS), mesenchymal stem cells (MSC), differentiation, metastasis

## Abstract

Rhabdomyosarcoma (RMS) is a soft tissue sarcoma, which may originate from impaired differentiation of mesenchymal stem cells (MSC). Expression of MET receptor is elevated in alveolar RMS subtype (ARMS) which is associated with worse prognosis, compared to embryonal RMS (ERMS). Forced differentiation of ARMS cells diminishes MET level and, as shown previously, MET silencing induces differentiation of ARMS. In ERMS cells introduction of TPR-MET oncogene leads to an uncontrolled overstimulation of the MET receptor downstream signaling pathways. *In vivo*, tumors formed by those cells in NOD-SCID mice display inhibited differentiation, enhanced proliferation, diminished apoptosis and increased infiltration of neutrophils. Consequently, tumors grow significantly faster and they display enhanced ability to metastasize to lungs and to vascularize due to elevated VEGF, MMP9 and miR-378 expression. *In vitro*, TPR-MET ERMS cells display enhanced migration, chemotaxis and invasion toward HGF and SDF-1. Introduction of TPR-MET into MSC increases survival and may induce expression of early myogenic factors depending on the genetic background, and it blocks terminal differentiation of skeletal myoblasts. To conclude, our results suggest that activation of MET signaling may cause defects in myogenic differentiation leading to rhabdomyosarcoma development and progression.

## INTRODUCTION

Rhabdomyosarcoma (RMS) is a soft-tissue sarcoma that is thought to originate from a defective differentiation of muscle progenitor cells or oncogenic transformation of mesenchymal stem cells [1]. It affects mainly children and adolescents and rarely occurs in adults. Based on histological and pathological features of RMS, two major subtypes can be distinguished: embryonal (ERMS) and alveolar (ARMS) [2]. ARMS tumor is usually associated with worse prognosis due to presence of t(2;13) or less common t(1;13) translocations, which result in presence of either PAX3-FKHR or PAX7-FKHR fusion genes [3].

High aggressiveness of ARMS subtype is also associated with increased levels of MET receptor, a member of tyrosine kinase receptors family (RTK) [2], [4]. The aberrant MET signaling is observed in many tumor types [5]. MET receptor responds to only one ligand – hepatocyte growth factor (HGF, also known as scatter factor, SF). MET receptor overexpression is one of the mechanisms that can induce tumor invasive behavior. Increased MET expression can originate from MET proto-oncogene amplification [6], enhanced transcription triggered by other oncogenes like RAS [7] and can be also induced by hypoxia [8]. Overexpressed receptors dimerize spontaneously and undergo activation even in the absence of HGF [6]. For example, excessive MET expression can be observed in some lung cancer cell lines [9] and may be used as a prognostic feature in prediction of primary colon cancer invasiveness [10].

Interestingly, MET receptor was first discovered as the product of TPR-MET oncogene in human osteogenic sarcoma cell line. The genetic translocation involves translocated promoter region (TPR) encoding leucine zipper motif and the MET kinase domain with sequence encoding C-terminus [11]. The fusion TPR-MET oncoprotein (65 kDa) locates in cytoplasm and is constitutively active due to the TPR leucine zipper interactions with MET kinase domain. This interaction is responsible for dimerization and ligand-independent oncogenic activity of TPR-MET [12]. As a consequence, the transgenic expression of TPR-MET can cause mammary tumors [13].

*In vivo*, HGF is produced mainly by cells of mesenchymal origin while MET receptor is expressed mostly on epithelial cells [14]. Nevertheless, cells of mesenchymal origin may acquire aberrant expression of MET receptor, what activates downstream MET signaling pathways and may lead to tumorigenesis [15,16]. This mechanism has been demonstrated for liposarcoma [17] or leiomyoma [18]. Increased level of the MET receptor in tumors of mesenchymal origin is usually associated with higher aggressiveness. For example in RMS tumors more aggressive ARMS cells display higher expression of MET than ERMS cell lines [19]. Increased level of the MET receptor in RMS may be explained by the defects in posttranscriptional regulation of its expression by miR-206 [20, 21].

Recently, we have demonstrated that downregulation of MET receptor in ARMS diminishes tumor growth, metastasis [22] and induces myogenic differentiation [23]. The aim of this study was to investigate which of the molecular mechanisms associated with constitutive activation of MET signaling are responsible for rhabdomyosarcoma development and malignancy. Therefore we constitutively activated MET signaling pathway in ERMS tumors, mesenchymal stem cells (MSC) and skeletal myoblasts by introduction of TPR-MET oncogene.

## RESULTS

### Activation of MET signaling pathways blocks myogenic differentiation of embryonal rhabdomyosarcoma and promotes tumor growth

**Figure 1:**
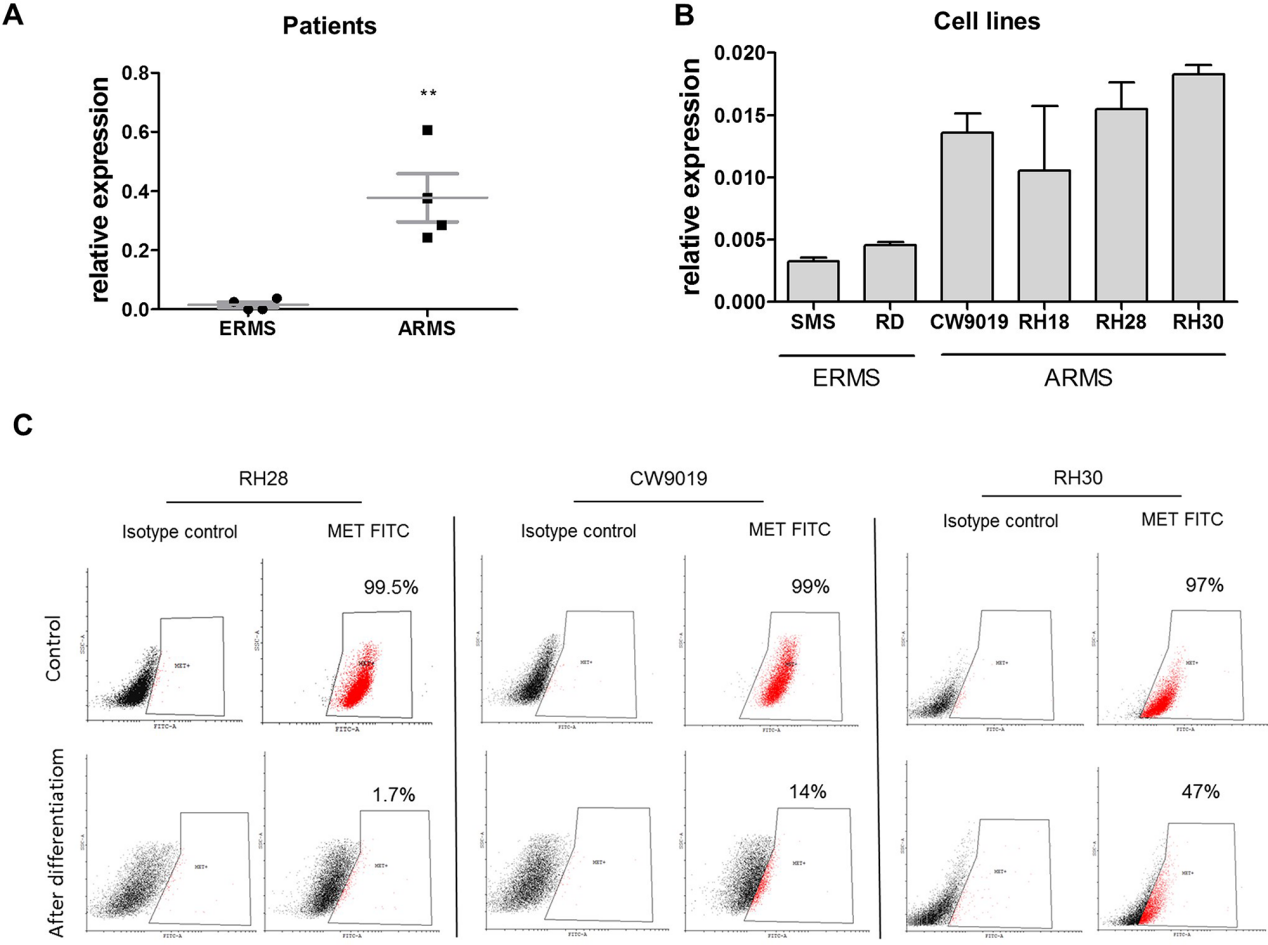
Expression of MET receptor is higher in ARMS than in ERMS and it decreases when ARMS cells are differentiated **A.** Relative expression of MET mRNA compared to GAPDH mRNA was evaluated by qPCR in samples isolated from paraffin embedded RMS specimens from patients; *n* = 4, **p < 0.01. **B.** Relative expression of MET mRNA compared to GAPDH mRNA was evaluated by qPCR in samples from RMS cell lines, *n* = 3. **C.** MET receptor level was evaluated by flow cytometry in RH28, CW9019 and RH30 ARMS cells undifferentiated and differentiated for 7–10 days in DMEM with 2% HS and TPA. Data show one representative experiment. Data in graphs are represented as mean +/− SEM.

Our previous study [19] and current analysis of new rhabdomyosarcoma tumor samples demonstrated that expression of MET mRNA is lower in embryonal (ERMS) than in alveolar (ARMS) subtype (Figure 1A), which is usually associated with more malignant phenotype [3]. Moreover, in those samples MET level positively correlated with MyoD expression (Supplementary Table 1), suggesting association of MET with a defect in myogenic differentiation in those tumors. Accordingly in SMS-CTR and RD ERMS cell lines expression of MET was lower than in CW9019, RH18, RH28 and RH30 alveolar cell lines (Figure 1B). We have previously demonstrated that downregulation of MET receptor in ARMS RH30 cell line induces myogenic differentiation [22]. *In vitro* differentiation of several ARMS cell lines such as RH30, CW9019 and RH28, diminished MET receptor level on the surface of the cells (Figure 1C). These results suggest that MET is an important factor in myogenic differentiation of RMS.

**Figure 2:**
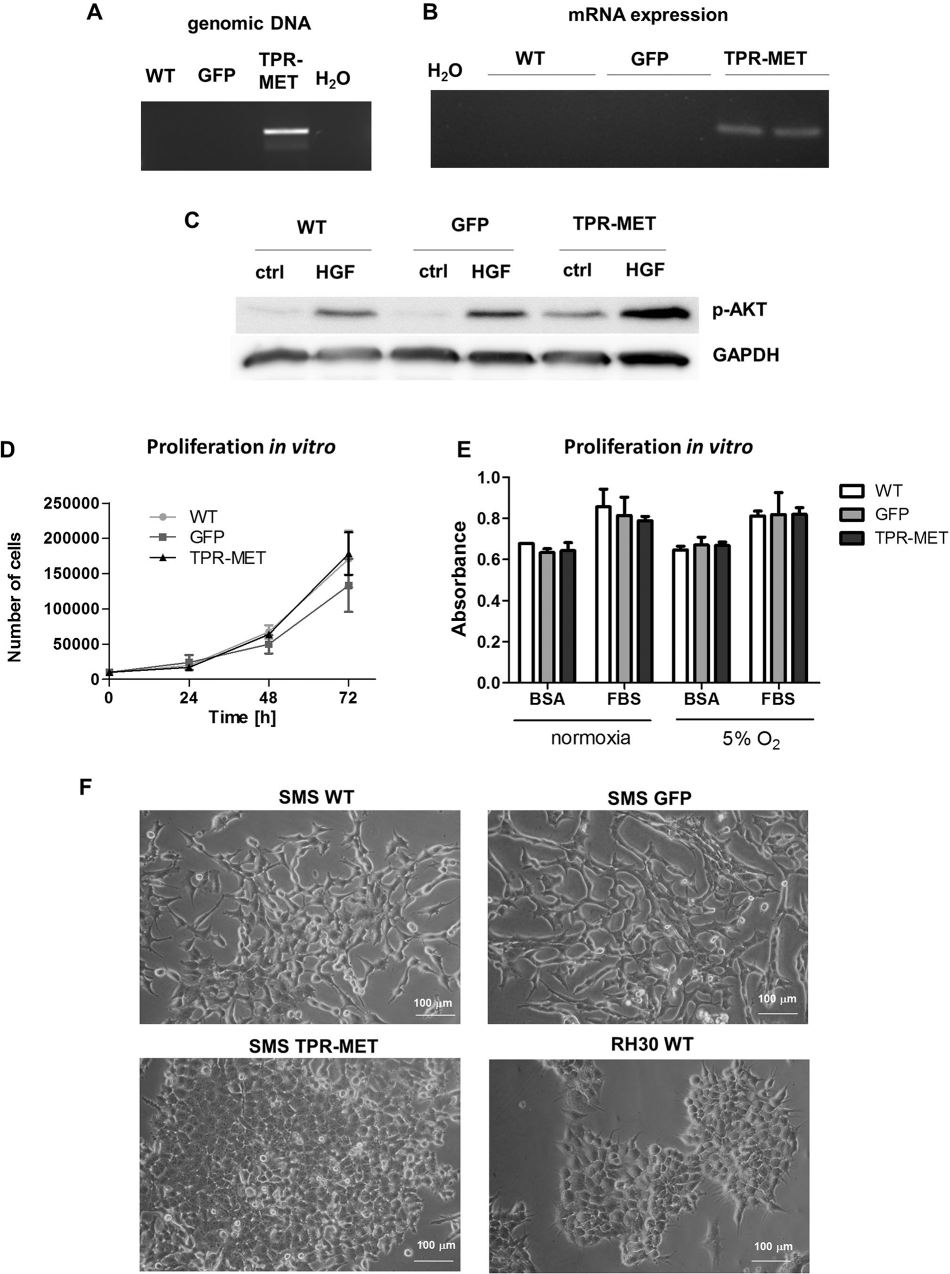
Introduction of TPR-MET oncogene to SMS-CTR ERMS cells constitutively activates downstream MET signaling pathways **A.** Incorporation of TPR-MET transgene to genomic DNA was validated by PCR. The picture shows one representative experiment. **B.** Expression of TPR-MET mRNA was estimated by PCR, *n* = 2. **C.** Increased phosphorylation of AKT kinases regardless of HGF (20 ng/ml) treatment in TPR-MET cells was demonstrated by Western blotting. The picture shows one representative experiment. **D.** Proliferation of the cells in standard cell culture conditions was calculated by counting of the cells in Burker chamber, *n* = 2. **E.** Proliferation was estimated with MTT assay in cells cultured in normoxia and at 5% O_2_ level, 10% FBS and 0.5% BSA, *n* = 2. **F.** TPR-MET SMS-CTR ERMS cells and RH30 ARMS cells form rounded colonies in Matrigel in medium supplemented with 2% HS in contrast to WT and GFP SMS-CTR cells which acquire an elongated phenotype. Data in graphs are represented as mean +/− SEM.

To determine if activation of MET signaling pathways may be responsible for activation of oncogenic and metastatic pathways in rhabdomyosarcoma development, we transduced SMS-CTR cells using lentiviral vectors harboring TPR-MET oncogene. TPR-MET was used as a model for constitutive activation of downstream MET signaling pathways independent of HGF ligand binding. We choose SMS-CTR cell line due to its low basal levels of MET receptor (Figure 1B). As controls we used SMS-CTR cells transduced with GFP. Development of stable cell lines was confirmed by incorporation of TPR-MET transgene to genomic DNA (Figure 2A) and by expression of TPR-MET mRNA (Figure 2B). Accordingly, in TPR-MET cells downstream MET signaling pathways were activated, as shown by constitutive phosphorylation of AKT kinases, regardless of HGF treatment. HGF also further potentiated phosphorylation of AKT kinases in TPR-MET cells (Figure 2C). Nevertheless, *in vitro* SMS-CTR cells with TPR-MET did not proliferate faster than control cells both in standard culture conditions (Figure 2D) and in starving conditions nor in hypoxia (Figure 2E). There was also no significant effect of TPR-MET on morphology of the cells. SMS-CTR cells poorly differentiated *in vitro*, when cultured in DMEM medium with 2% horse serum (data not shown). However, when those cells were cultured in Matrigel in medium supplemented with 2% horse serum, significant morphological changes appeared. Control cells acquired elongated shape, whereas SMS TPR-MET cells formed rounded colonies, which resembled colonies, formed by ARMS RH30 cells displaying high basal MET level (Figure 2F).

**Figure 3:**
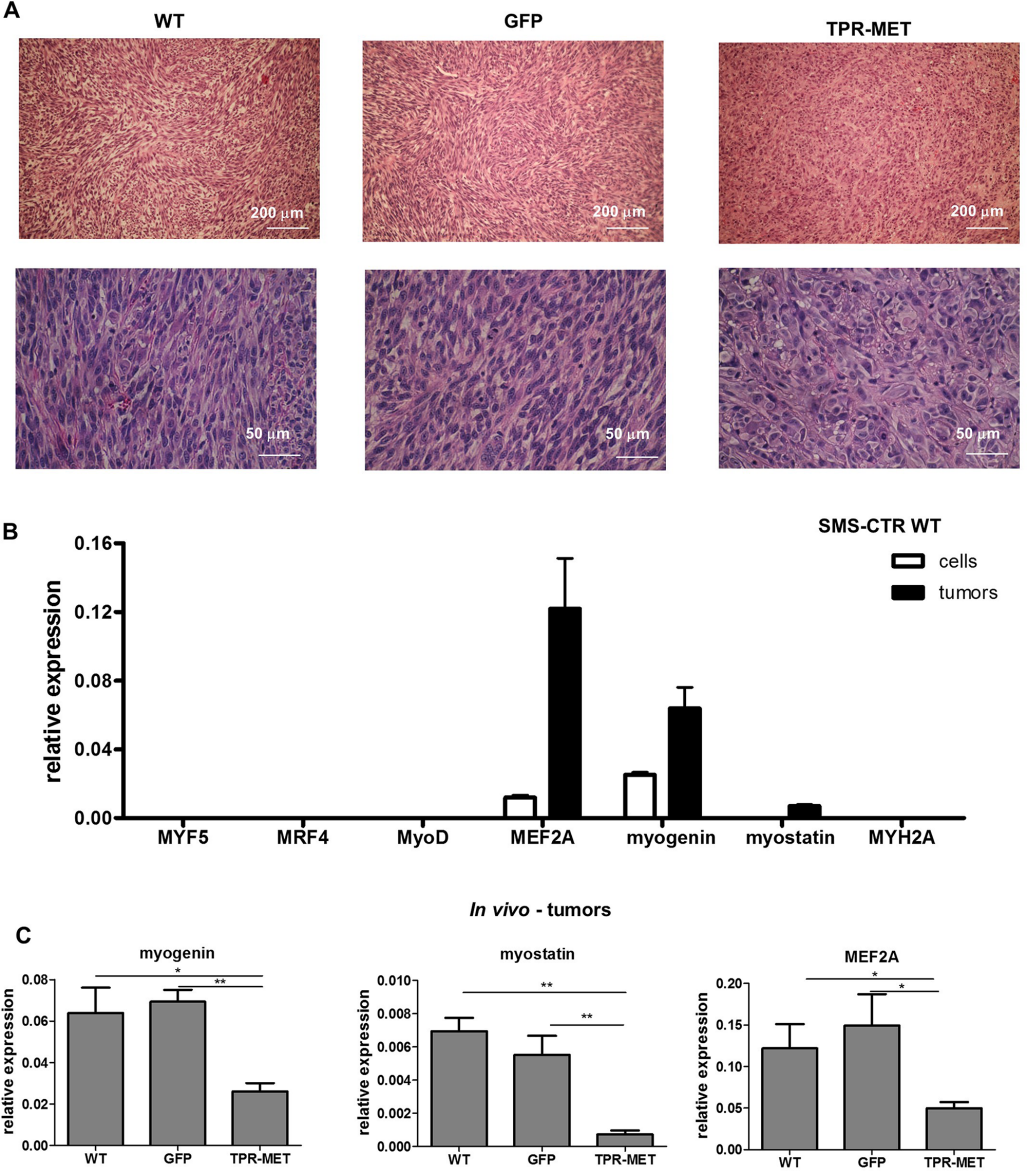
Activation of MET signaling in SMS-CTR ERMS blocks myogenic differentiation of tumors *in vivo* **A.** Hematoxylin-eosin staining showed anaplastic morphology of tumors formed by SMS-CTR cells expressing TPR-MET four weeks after subcutaneous implantation of the cells to immunodeficient NOD-SCID mice, whereas control tumors were formed by cells resembling muscle fibers. **B.** Expression of myogenic differentiation markers was evaluated at mRNA level by qPCR in SMS-CTR wild-type cells and in paraffin-embedded specimens from tumors formed by SMS-CTR cells after subcutaneous implantation of the cells, *n* = 4–5. **C.** Constitutive activation of MET signaling in SMS-CTR tumors in NOD-SCID mice inhibits myogenin, myostatin and MEF2A level, qPCR, *n* = 4–5. *p < 0.05, **p < 0.01. Data in graphs are represented as mean +/− SEM.

To investigate this phenomenon further, *in vivo* experiments were performed. Subcutaneous implantation of the cells into NOD-SCID immunodeficient mice results in their differentiation. The tumor cells acquire a spindle shape, which is a feature characteristic for muscle fibers (Figure 3A). This effect coincides with upregulation of factors regulating myogenic differentiation, such as myocyte enhancer factor 2A (MEF2A), myogenin and myostatin in tumors *in vivo* compared to cells *in vitro* (Figure 3B). Similar effect is seen in SMS-CTR cells cultured in DMEM medium with 2% horse serum, but it is less potent (Supplementary Figure 1). Interestingly, in rhabdomyosarcoma samples, MEF2A level positively correlated with myogenin expression, whereas myogenin level also positively correlated with myosin heavy chain 2 (MYH2A), a marker of late differentiation (Supplementary Table 1). In our model constitutive activation of MET signaling pathways blocked differentiation of the tumor cells *in vivo*, as TPR-MET tumors were formed by pleomorphic cells that did not shape into muscle fiber-like structures and were characterized by more undifferentiated morphology (Figure 3A). This effect may be explained by inhibition of expression of myogenic markers, such as MEF2A, myogenin and myostatin in tumors formed by SMS-CTR cells with constitutively active MET signaling (Figure 3C). It also turned out that this effect is independent of myomiRs, such as miR-1, miR-133a, miR-133b and miR-206, as TPR-MET did not regulate them *in vivo* nor *in vitro* (Supplementary Figure 2A and 2B).

**Figure 4:**
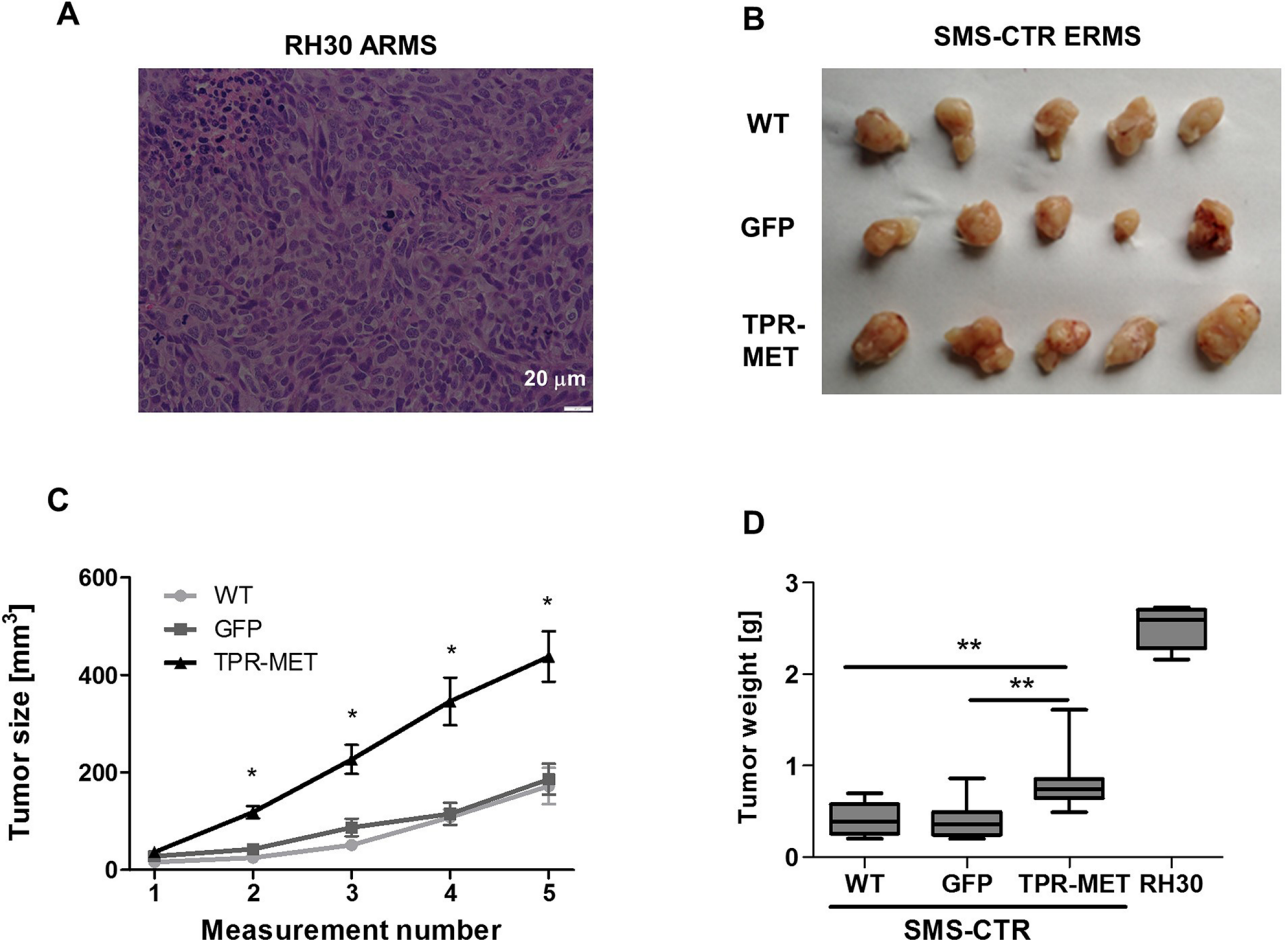
Activation of MET signaling in SMS-CTR ERMS cells enhances tumor growth *in vivo* **A.** Hematoxylin-eosin staining showed anaplastic morphology of tumors formed by RH30 ARMS cells with high basal MET level after subcutaneous implantation of the cells to immunodeficient NOD-SCID mice. **B.** Photograph of tumors formed by SMS-CTR cells isolated four weeks after subcutaneous implantation of the cells into NOD-SCID mice shows their differences in size. **C.** Tumor size was measured with caliper in different time points, *n* = 7–9. **D.** SMS-CTR and RH30 tumor weight was evaluated at the end of experiment, *n* = 4–9. Data in graphs are represented as mean +/− SEM.

**Figure 5:**
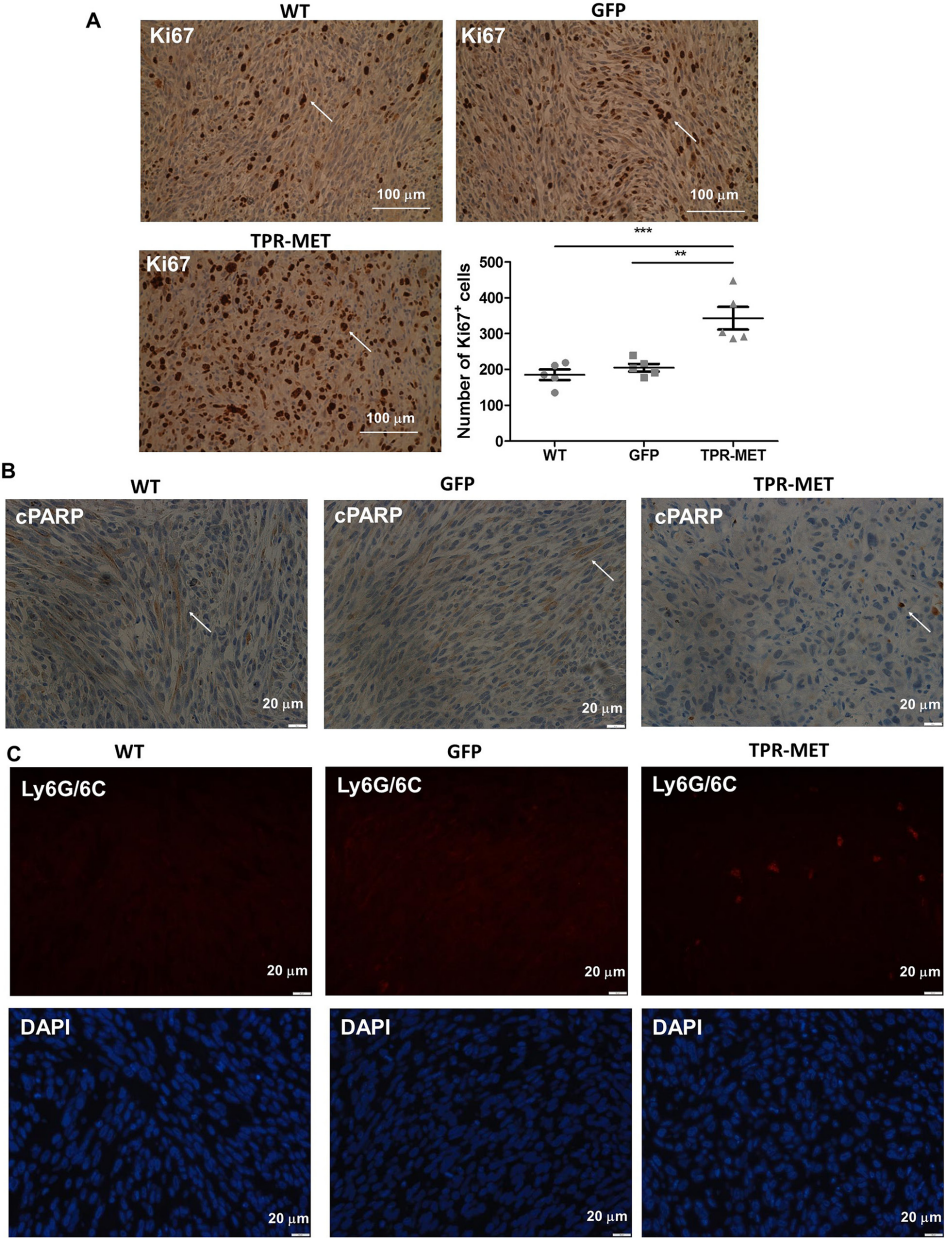
Activation of MET signaling in SMS-CTR ERMS cells enhances tumor proliferation, decreases tumor apoptosis and induces infiltration of neutrophils *in vivo* **A.** Representative images of the staining for Ki67 in tumor sections show areas of tumor cells proliferation. Number of Ki67 positive cells was calculated in non-necrotic areas of tumor specimens, *n* = 5. Arrows indicate Ki67 positive cells. **B.** Representative images of the staining for cleaved PARP demonstrate decreased apoptosis in TPR-MET SMS-CTR tumors. **C.** Representative images of the staining for neutrophils (Ly6G/6C) show infiltration of murine neutrophils to TPR-MET SMS-CTR tumors. *p < 0.05, **p < 0.01, ***p < 0.001. Data in graphs are represented as mean +/− SEM. Arrows indicate cPARP positive cells.

Anaplastic morphology of TPR-MET ERMS tumors resembled the morphology of ARMS tumors formed by RH30 cells displaying high basal MET level (Figure 4A). Tumors with the constitutively activated MET signaling were not only less differentiated than control ERMS tumors, but they were also bigger (Figure 4B). They grew faster (Figure 4C) and after four weeks they weighed more than control tumors (Figure 4D) but less than tumors formed by ARMS RH30 cells displaying high basal MET level, probably because other genes besides MET are also responsible for higher ARMS invasiveness. The effect of TPR-MET on tumor growth coincided with enhanced proliferation, what was demonstrated by staining for Ki67 in non-necrotic areas of tumors sections (Figure 5A). The staining also revealed that the areas in control tumors with the elongated cells resembling muscle fibers displayed decreased proliferation (Figure 5A). The opposing effect was visible for the apoptotic cells. Staining for cleaved PARP (cPARP) revealed its presence in more elongated control cells (Figure 5B). Enhanced growth of TPR-MET tumor may be explained not only by the increased proliferation and decreased apoptosis, but also by infiltration of murine neutrophils to those tumors, what is demonstrated by Ly6G/6C staining of tumor sections (Figure 5C). What is also interesting, due to enhanced growth rate, TPR-MET tumors displayed also big necrotic areas in the central region of tumors (Supplementary Figure 3). Those results suggest that the enhanced tumor growth may be associated with inhibition of differentiation *in vivo* and with additional signals provided by tumor microenvironment. Accordingly, our previous studies demonstrated that RH30 ARMS cells with silenced MET level displayed diminished tumor growth and metastasis [22, 23].

### Activation of MET signaling pathways induces angiogenesis, migration and metastasis of rhabdomyosarcoma

**Figure 6:**
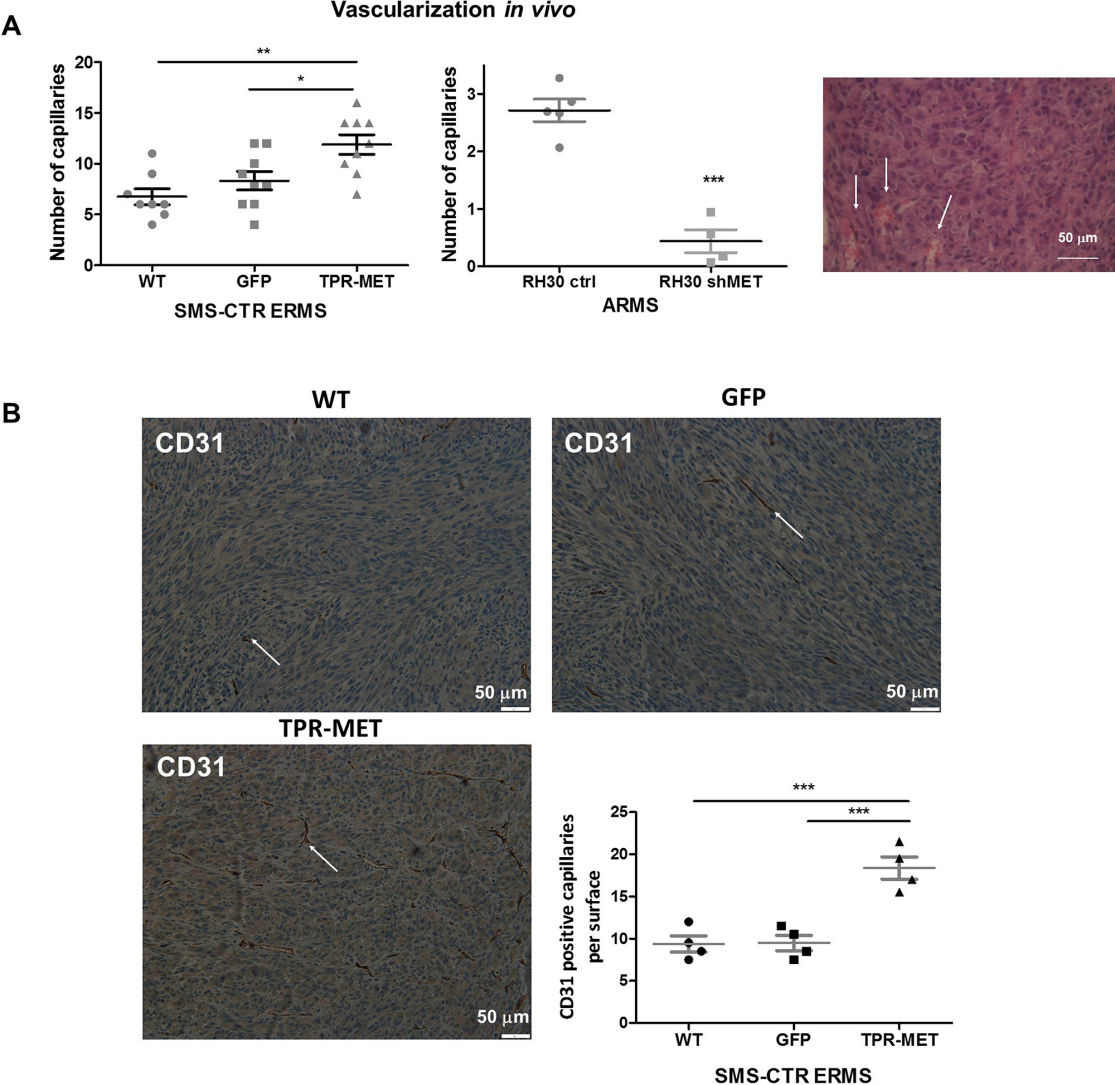
Activation of MET signaling in SMS-CTR ERMS enhances tumor vascularization *in vivo*, whereas MET silencing in RH30 ARMS exerts the opposite effects **A.** Activated MET signaling increases number of capillaries in tumors formed by SMS-CTR ERMS and RH30 ARMS cells after subcutaneous implantation to NOD-SCID mice. Capillaries with erythrocytes were counted after staining of tumor sections for hematoxylin-eosin, *n* = 7–9. **B.** Representative images of the staining for CD31 demonstrates increased number of capillaries in TPR-MET SMS-CTR tumors *in vivo*. Number of CD31 positive capillaries was calculated in non-necrotic areas of tumor specimens, *n* = 4. *p < 0.05, **p < 0.01, ***p < 0.001. Data in graphs are represented as mean +/− SEM. Arrows indicate the capillaries.

Hematoxylin-eosin staining of paraffin-embedded tumor sections enabled to visualize blood vessels containing erythrocytes. It turned out that constitutive activation of MET signaling in SMS-CTR cells promoted development of capillaries inside tumors, whereas in tumors formed by ARMS RH30 cells with decreased MET expression (RH30 shMET) after subcutaneous implantation into NOD-SCID mice the number of capillaries was diminished compared to control RH30 cells (Figure 6A). Staining for CD31 – a marker of blood vessels additionally confirmed that TPR-MET tumors display higher number of CD31-positive capillaries compared to control tumors (Figure 6B).

**Figure 7:**
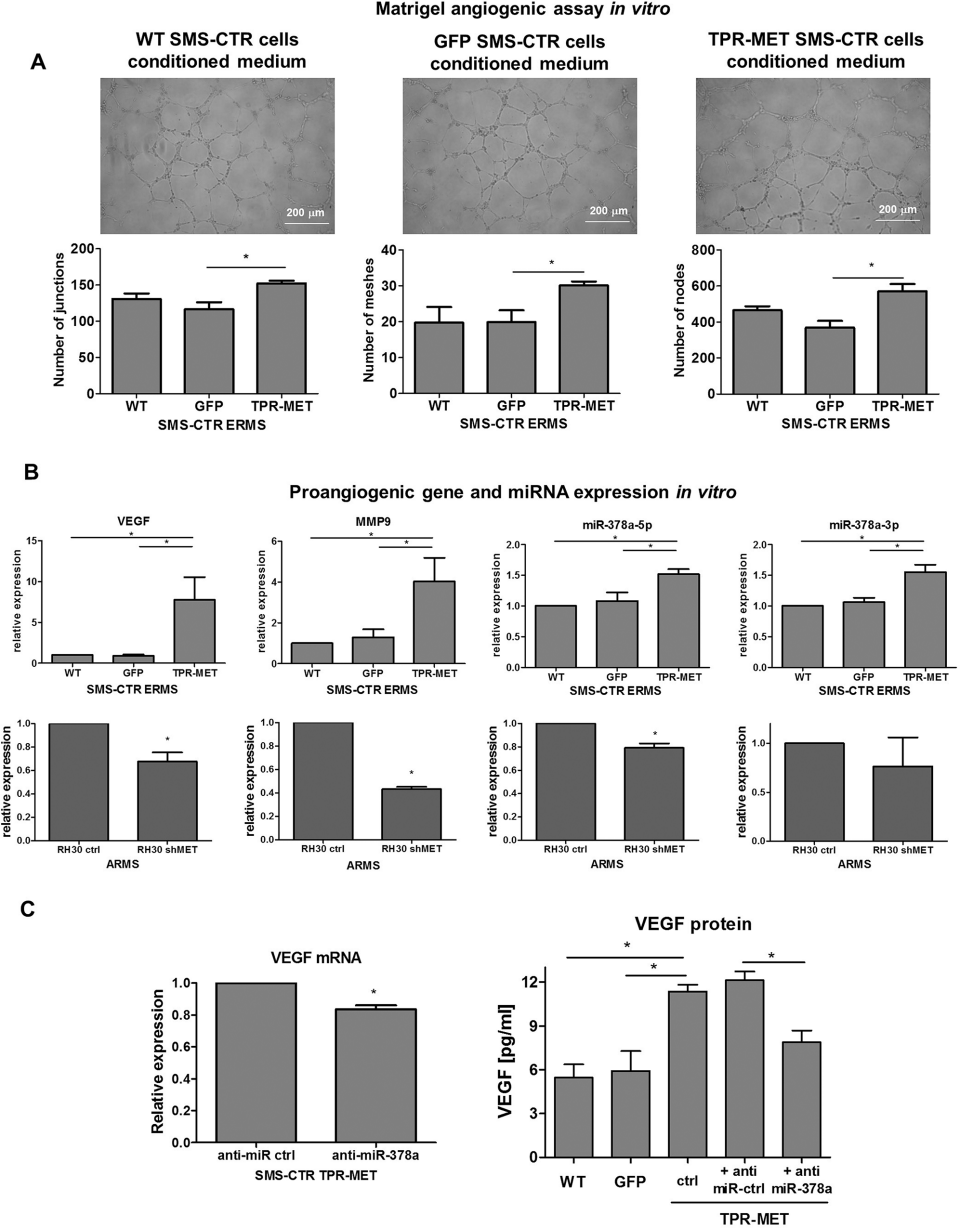
Activation of MET signaling in SMS-CTR ERMS cells induces proangiogenic effects *in vitro* by upregulation of miR-378, VEGF and MMP9, whereas MET silencing in RH30 ARMS exerts the opposite effects **A.** TPR-MET SMS-CTR cells conditioned media increase the amount of junctions, nodes and meshes formed by HUVEC cells in Matrigel angiogenesis assay *in vitro*, *n* = 4. **B.** Expression of VEGF, MMP9, miR-378a-5p and miR-378-3p is increased in SMS-CTR ERMS cells expressing TPR-MET and downregulated in shMET RH30 ARMS cells *in vitro*, qPCR, *n* = 2–4. **C.** Inhibition of miR-378a with anti-miR sequences reverses the effect of TPR-MET on VEGF mRNA and protein, *n* = 2–4. *p < 0.05, **p < 0.01, ***p < 0.001. Data in graphs are represented as mean +/− SEM.

Similarly, *in vitro* TPR-MET SMS-CTR cells conditioned media increased the number of junctions, nodes and meshes formed by HUVEC cells in Matrigel angiogenic assay (Figure 7A). Those proangiogenic effects may be explained by enhanced expression of miR-378a, MMP9 and VEGF in SMS-CTR cells expressing TPR-MET, whereas antiangiogenic capabilities of ARMS cells with silenced MET level may be explained by decreased expression of those factors (Figure 7B). Moreover, inhibition of miR-378a with anti-miR-378a inhibitor reversed the effect of TPR-MET on VEGF mRNA and protein level (Figure 7C). Those results demonstrate for the first time that one of the proangiogenic mediators of the MET action may be miR-378.

**Figure 8:**
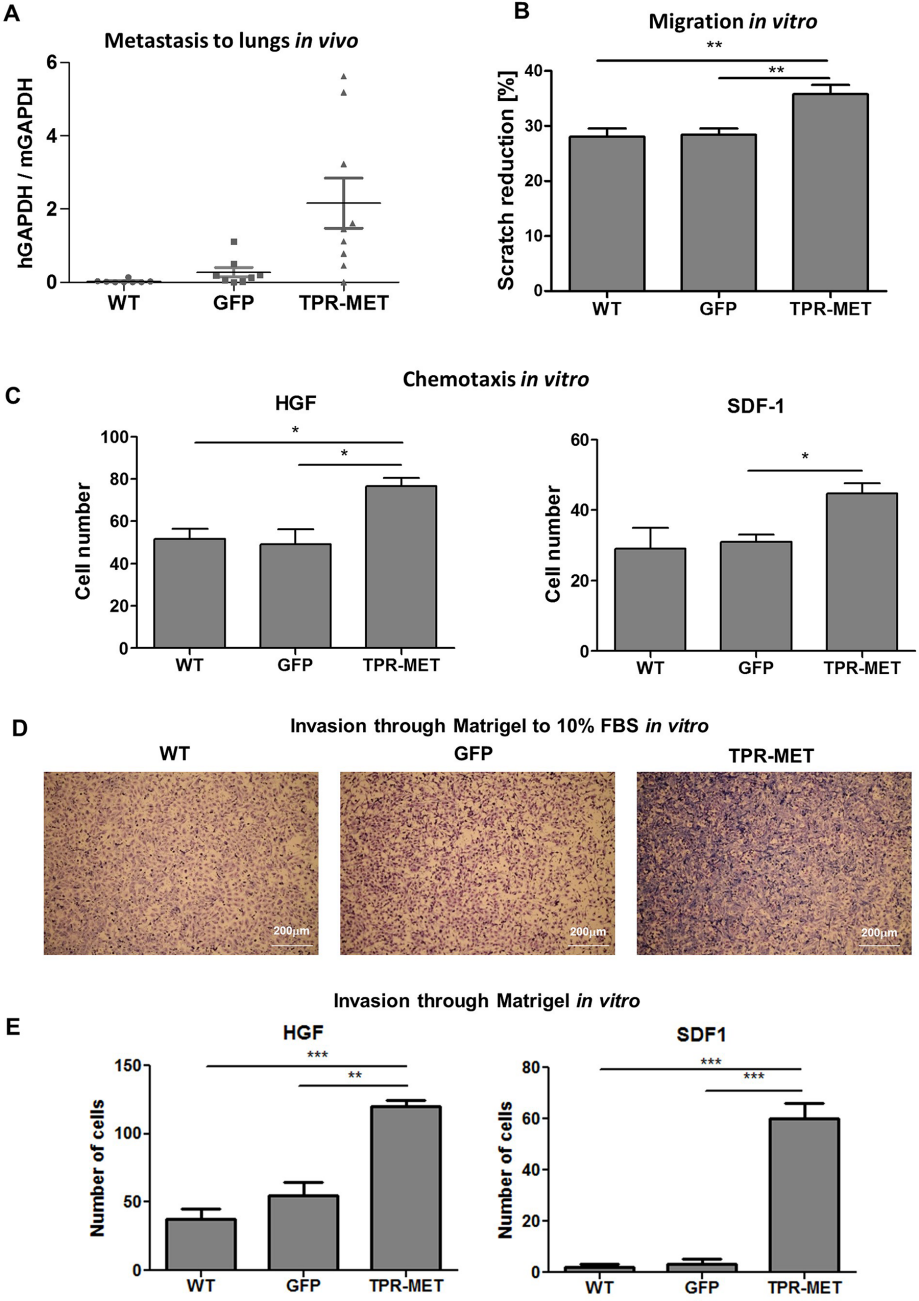
Activation of MET signaling in SMS-CTR ERMS induces metastasis to lungs *in vivo* and enhances migratory and invasive capabilities of the cells *in vitro* **A.** Enhanced metastasis to lungs of SMS-CTR subcutaneous xenotransplants in NOD-SCID mice was demonstrated by evaluation of human GAPDH to murine GAPDH mRNA ratio by qPCR, *n* = 7–9. **B.** TPR-MET expressing cells closed the gap in a scratch assay slightly faster, *n* = 3. **C.** In a chemotactic assay TPR-MET expressing cells displayed enhanced migration toward HGF (20 ng/ml) and SDF-1 (100 ng/ml), *n* = 3. **D.** Representative images of invasion of SMS-CTR cells through Matrigel to 10% FBS *in vitro* show enhanced invasive capabilities of TPR-MET cells. **E.** TPR-MET SMS-CTR cells display enhanced invasion through Matrigel to HGF and SDF1, *n* = 3. *p < 0.05, **p < 0.01, ***p < 0.001. Data in graphs are represented as mean +/− SEM.

Enhanced vascularization of TPR-MET tumors was accompanied by the induction of metastasis to lungs (Figure 8A). Higher metastatic potential may be explained by the enhanced migratory capabilities of SMS-CTR cells, what was shown in a scratch assay *in vitro* – migration in starving conditions in medium with 0.5% BSA was enhanced in TPR-MET cells (Figure 8B). Due to enhanced migratory capabilities those cells displayed also enhanced chemotaxis toward gradients of both human HGF and SDF-1 *in vitro* (Figure 8C). Not only chemotaxis, but also invasion through Matrigel to FBS (Figure 8D), HGF and SDF-1 (Figure 8E) *in vitro* was strongly enhanced in TPR-MET cells.

### Activation of MET signaling in mesenchymal stem cells induces prolonged survival and may drive an oncogenic transformation, and it blocks terminal differentiation of skeletal myoblasts

**Figure 9:**
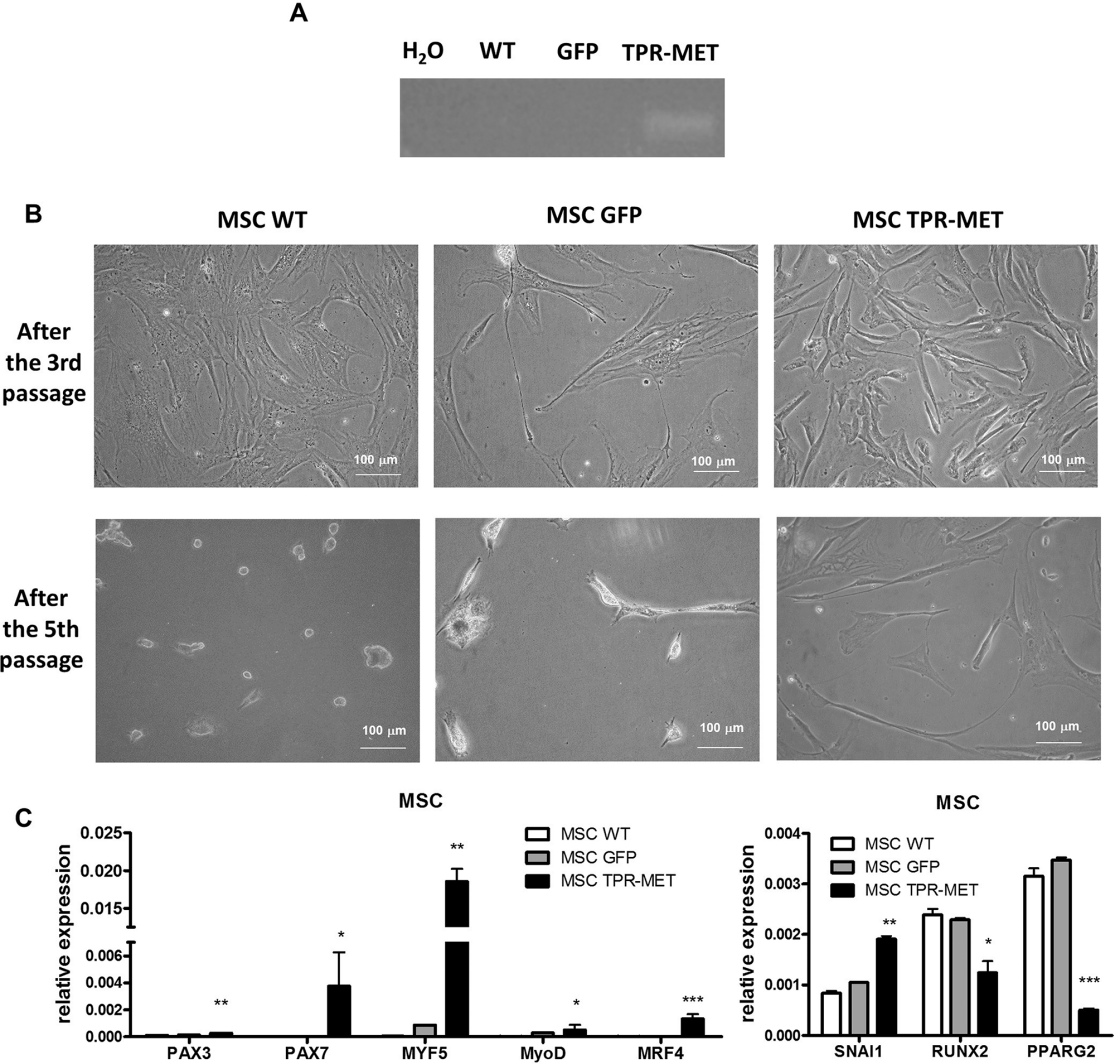
Activation of MET signaling in human mesenchymal stem cells induces prolonged survival and expression of myogenic markers **A.** MSC were transduced with viral vectors encoding GFP and TPR-MET and selected with blasticidin. Expression of TPR-MET transgene at mRNA level was verified by PCR. The picture shows one representative experiment. **B.** Representative photos show morphology of MSC after 3 and 5 passages. After 5 passages control MSC are senescent, whereas TPR-MET MSC still grow. **C.** When TPR-MET MSC were cultured for several months, expression of myogenic factors, such as PAX3, PAX7, MYF5, MyoD and MRF4, was induced, whereas expression of osteoblastic RUNX2 and adipogenic PPARG2 factors decreases, what coincided with upregulation of SNAI1, qPCR, *n* = 2. *p < 0.05. Data in graphs are represented as mean +/− SEM.

**Figure 10:**
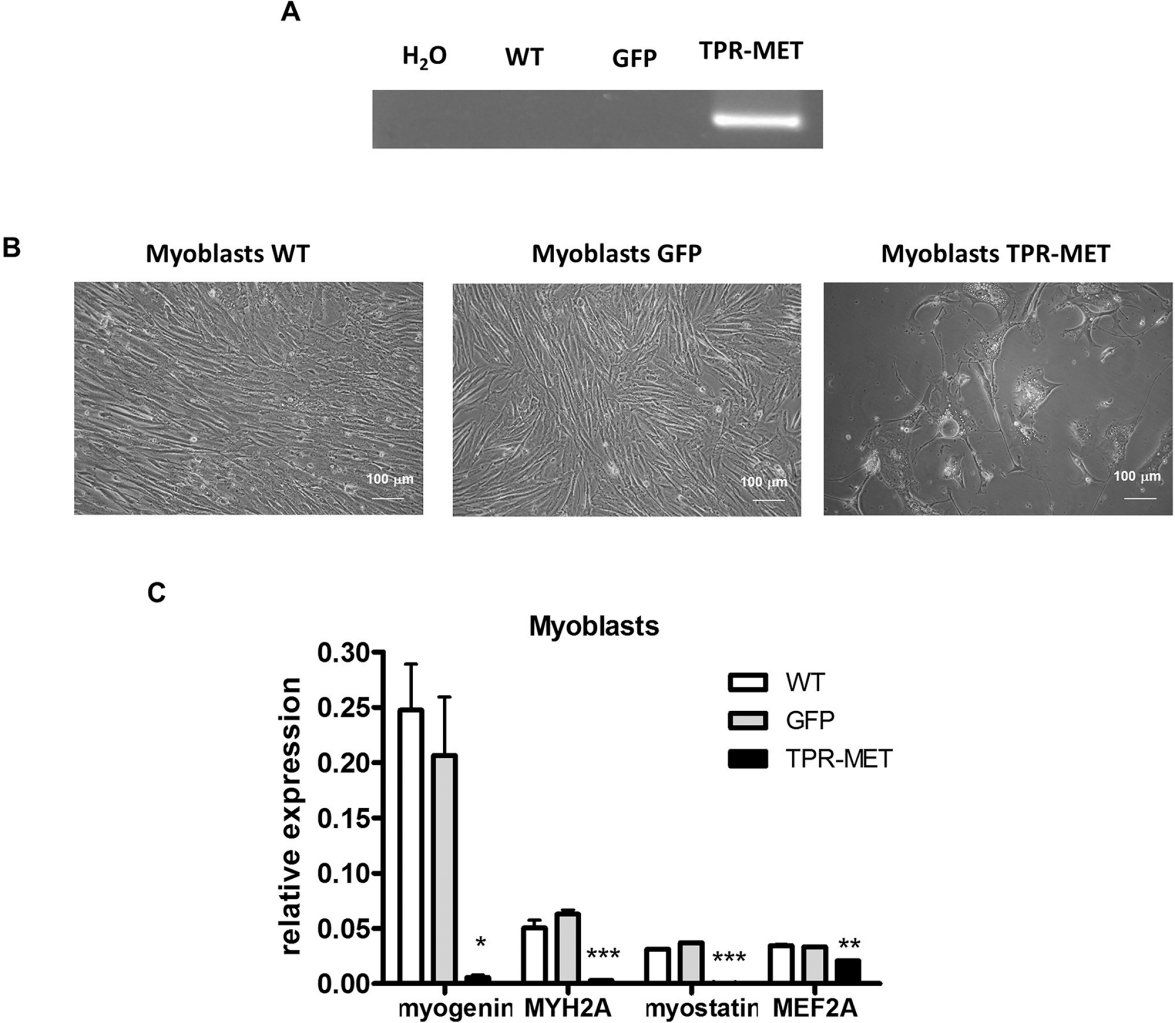
Activation of MET signaling in human skeletal myoblasts blocks their growth and terminal differentiation **A.** Human skeletal myoblasts were transduced with viral vectors encoding GFP and TPR-MET and selected with blasticidin. Expression of TPR-MET transgene at mRNA level was verified by PCR. The picture shows one representative experiment. **B.** Representative photos show morphology of human myoblasts after 4 passages. Introduction of TPR-MET induces cytotoxicity, blocks myoblasts growth and terminal differentiation. **C.** TPR-MET myoblasts display decreased expression of late myogenic factors, such as myogenin, myosin heavy chain, myostatin and MEF2A, qPCR, *n* = 2. *p < 0.05, **p < 0.01. Data in graphs are represented as mean +/− SEM.

Because one of the hypotheses underlying rhabomyosarcoma development states that it may originate from a differentiation defect of mesenchymal stem cells (MSC), we introduced TPR-MET with viral vectors into MSC to constitutively activate MET signaling pathways (Figure 9A). Control cells were modified with vector harboring GFP. Positive cells were selected with blasticidin and cultured for several passages. Control cells survived in culture only until the 5^th^ passage and then they started to die, whereas TPR-MET MSC survived in culture for more than 5 passages (Figure 9B). Surprisingly, in MSC expressing TPR-MET, which survived in culture for several months, we observed an induction of expression of myogenic markers, such as PAX3, PAX7, MYF5, MyoD and MRF4 (Figure 9C). MYF5 was the most potently upregulated early myogenic factor. Expression of osteoblastic RUNX2 and adipogenic PPARG2 negatively correlated with SNAI1 expression (Figure 9C). Those results suggest that MSC cells expressing MYF5 and PAX7 started to differentiate into muscle progenitors. Nevertheless, there was no spontaneous differentiation towards myoblasts and mature muscle fibers, what suggests that those cells were kept in an early stage of myogenic differentiation. On the other hand, introduction of TPR-MET to human myoblasts (Figure 10A) changed their morphology, induced cytotoxicity and blocked formation of mature myotubes (Figure 10B), what coincided with inhibition of expression of late myogenic factors, such as myostatin, myogenin, myosin heavy chain and also MEF2A (Figure 10C). Those results indicate that for proper terminal differentiation of myoblasts activation of MET should be diminished. Our results suggest that constitutive activation of MET signaling induces prolonged survival of MSC *in vitro* and may induce early myogenic differentiation. Nevertheless, in the future more detailed studies are required to demonstrate the mechanisms of that process.

## DISCUSSION

The salient finding of the present study is that constitutive activation of MET signaling by expression of TPR-MET oncogene induces rhabdomyosarcoma development by blocking myogenic differentiation and enhancing proliferation, migration and angiogenesis. We have chosen TPR-MET as a model of constitutive activation of MET downstream signaling pathways, which is independent of HGF ligand binding. Our group [22] and others [4] have previously shown that downregulation of MET receptor diminishes RMS growth and promotes myogenic differentiation of the tumor. In development of skeletal muscles MET receptor plays a similar role. Constitutive activation of MET signaling by introduction of TPR-MET leads to myotube breakdown and muscle atrophy [24]. It is in accordance to our studies: activation of MET signaling in differentiating human myoblasts was cytotoxic, what indicates that in proper terminal myogenic differentiation it should be diminished. Moreover, HGF and MET are co-localized in activated satellite cells in regions of muscle repair [25]. When myoblasts stop proliferation and differentiate both MET receptor and HGF are downregulated. Introduction of either TPR-MET or both HGF and MET receptor inhibits myogenesis at both morphological and biochemical levels, as myogenin and MyoD levels are diminished [26]. Similarly, in our current studies we demonstrated that differentiation of ARMS decreases MET receptor levels. In RMS tumors expressing TPR-MET we also observed downregulation of myogenic markers, such as myogenin, myostatin and MEF2A, what explains morphological changes in those tumors. The effects of TPR-MET are also independent of myomiRs, miRNAs regulating myogenesis, which have previously been demonstrated to regulate rhabdomyosarcoma development [27].

The significant role of MET signaling pathway in RMS differentiation was observed in experiments *in vivo*, whereas *in vitro* SMS-CTR cells poorly differentiated. One of the crucial factors responsible for differentiation *in vivo* may be three dimensional tumor microenvironment. When SMS-CTR cells were cultured in a differentiating medium in Matrigel instead of conventional two dimensional cell culture on plastic dishes, strong effect on morphological features was observed. Control SMS-CTR cells became more elongated, whereas TPR-MET cells formed rounded colonies similar to colonies formed by RH30 ARMS cells, which display higher basal MET level. The morphology of those cells resembled the morphology of the cells forming tumors in mice. It has been previously shown that RMS differentiate in spheroids and accumulate the differentiated myotube-like cells in the center [28]. Another important factor affecting tumor growth and differentiation is tumor microenvironment. Murine neutrophils infiltrating TPR-MET tumors may modulate both processes. Tumor-associated neutrophils are usually recruited by tumor in response to IL-8 and they have been previously shown to function against the host and usually they confer a poor prognosis, because they may promote enhanced metastasis and vascularization [29]. However, MET receptor is expressed not only by cancer cells but also by tumor-associated stromal cells and it is required for the recruitment of anti-tumor neutrophils in response to HGF. It has been previously shown that MET deletion in mouse neutrophils enhances tumor growth and metastasis [30].

Block of differentiation of RMS tumors by constitutive activation of MET receptor signaling may explain an enhanced proliferation of the tumors, what was demonstrated by staining for Ki67 and observation of tumor growth. Similarly, in satellite cells MET activation inhibits the exit from the cell cycle and delays myogenic differentiation [25]. Accordingly MET activation diminished apoptosis of tumor cells *in vivo*, as demonstrated by staining for cleaved PARP. Proliferation, cell motility and cell survival are regulated by MET *via* AKT pathway [31]. Aberrant phosphorylation of AKT kinases was observed in SMS-CTR tumors expressing TPR-MET. Constitutive activation of MET signaling pathway led to formation of tumors with morphology resembling ARMS. Nevertheless, ARMS tumors were bigger, probably because besides MET also other crucial genes may play a role in higher invasiveness, such as PAX3-FKHR [3].

In both physiological and pathological conditions MET regulates not only proliferation, but also migration of the cells toward HGF gradient. In MET deficient mice myogenic precursors are unable to migrate from the somites to the limbs [32]. Similarly, in pathological RMS conditions MET receptor downregulation decreases ability of ARMS cells to metastasize [23]. The metastatic behavior of RMS cells is regulated by both HGF and SDF-1 [33]. Our current studies revealed that constitutive activation of MET signaling increased metastasis to lungs in murine model, probably due to enhanced migratory capabilities of ERMS cells. *In vitro*, chemotaxis and invasion through Matrigel to both human HGF and SDF-1 were also elevated. Increased chemotaxis of the tumor cells with activated MET signaling may be relevant to RMS metastasis in patients.

Tumor progression and dissemination requires the development of new blood vessels [34]. In TPR-MET ERMS tumors we observed an enhanced vascularization, whereas in ARMS with decreased MET level the effect was opposite. One of the proangiogenic mediators in this process may be VEGF. Upregulation of VEGF by HGF-MET has been demonstrated in previous studies in different cell types [11]. We hypothesize that miR-378 may be one of the mediators of proangiogenic and oncogenic actions of MET. miR-378 can promote VEGF expression by competing with miR-125a for the same seed region in the VEGF 3′UTR [35]. In our studies inhibition of miR-378a with anti-miR sequences reversed the effect of TPR-MET on VEGF level. miR-378 has been previously demonstrated to induce progression and vascularization of many tumor types, both of epithelial and mesenchymal origin, such as, glioblastoma [36], non-small cell lung carcinoma [37] or breast cancer [38]. In case of sarcomas miR-378* has been shown to be downregulated in osteosarcoma tumor compared to normal osteoblasts [39]. In contrary, in rhabdomyosarcoma tumors miR-378 family members were demonstrated to be downregulated [40] and miR-378 was shown to induce myogenic differentiation by increasing the transcriptional activity of MyoD, in part by repressing an antagonist MyoR [41]. In our studies we have observed only the influence of miR-378 on tumor vascularization and no effect on myogenic differentiation, probably because our SMS-CTR cell model does not display high MyoD expression. Our studies revealed also that MMP9 may be an important mediator of the MET signaling pathway in RMS development. MMP9 plays a central role inangiogenesis, stromal remodeling, and consequently metastasis of different tumor types [42].

RMS is a soft tissue tumor, which derives either from a defective differentiation of muscle progenitor cells or mesenchymal stem cells (MSC) [1]. It has been shown previously that expression of TPR-MET in differentiating muscles resulted in muscle wasting, but did not cause development of musculoskeletal tumors [24]. Our current studies demonstrated that introduction of MET induces prolonged survival of MSC in cell culture *in vitro*. Moreover, in those cells we detected expression of early myogenic factors, such as PAX7, MYF5, MyoD and MRF4. Analysis of a hierarchy of transcription factors regulating progression through the myogenic lineage [43] revealed that those cells resembled rather satellite stem cells due to the high level of early myogenic markers, such as MYF5 and PAX7. However, we did not observe any spontaneous differentiation of those cells into myoblasts or mature myofibers. This suggests that constitutive activation of MET signaling may drive an oncogenic transformation toward sarcoma tumors, but the effect might be dependent on genetic background. The hypothesis is strengthen by the fact that MSC exposed to pro-longed treatment with HGF start to express the markers of the first stages of muscle differentiation, whereas they concomitantly loose stem cell markers [44]. On the other hand, silencing of MET with shRNA impairs MSC differentiation into the osteoblastic and chondrogenic lineages [45]. In our studies, concomitantly with upregulation of myogenic markers, we also observed downregulation of osteoblastic RUNX2 and adipogenic PPARG2 marker, probably due to upregulation of SNAI1. SNAI1 has been previously shown to be a regulator of osteoblastic and adipogenic differentiation. MSC cells with silenced SNAI1 level prematurely differentiate to osteoblasts or adipocytes [46]. During osteoblast differentiation SNAI1 regulates RUNX2 expression [47], whereas during adipocyte differentiation SNAI1 is a regulator of PPARγ expression [48]. Our current studies suggest that rhabdomyosarcoma may derive from the impaired differentiation of mesenchymal stem cells. We have demonstrated for the first time that constitutive activation of MET signaling pathway induces early myogenic differentiation of MSC. However, for development of skeletal muscles and late steps of differentiation this signaling needs to be downregulated. We suggest that impairment in those differentiation steps may lead to RMS development. This hypothesis is additionally supported by the fact that transgenic mice overexpressing HGF are also predisposed to develop RMS with high expression of MET receptor and elevated MET kinase activity [49, 50]. Those results strongly suggest that autocrine MET signaling broadly promotes RMS tumorigenesis. The statement is additionally supported by the fact that HGF-MET axis maintains cancer stem cells functions in different tumor types, such as glioblastoma [51], head and neck squamous cell carcinoma [52], pancreatic [53], colon cancer [54] or cervical carcinoma [55]. However, additional, more detailed studies are required to demonstrate the precise mechanism of RMS development. It has to be taken into consideration that this effect might be strongly dependent on genetic background of the cells. The cooperation of TPR-MET with the existing mutations may be crucial for induction of myogenic differentiation of MSC and possible interactions should be investigated in the future. This hypothesis is additionally supported by previous studies on MSC tumorigenesis. For example it has been shown previously that mouse mesenchymal stem cells expressing PAX3-FKHR fusion gene form alveolar rhabdomyosarcomas by cooperating with secondary mutations [56]. There is also an evidence that deficiency of different cell cycle regulators, such as p53, may trigger a transformation process in mouse MSC resulting in the generation of sarcoma [57].

**Figure 11:**
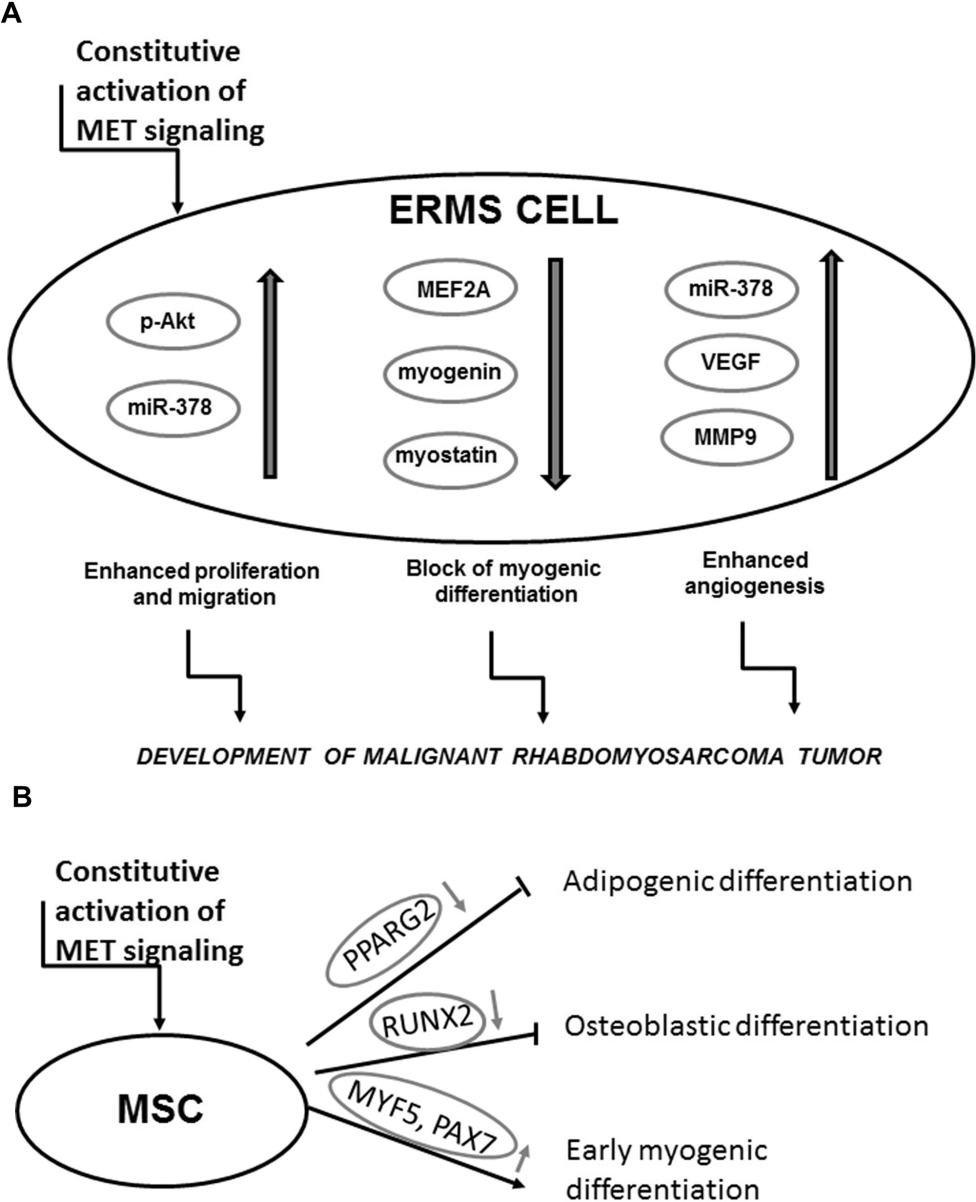
Activation of MET signaling blocks myogenic differentiation and promotes rhabdomyosarcoma development, angiogenesis and malignancy **A.** Mechanism of a development of malignant tumor by the constitutive activation of the MET receptor signaling in ERMS. **B.** Mechanism of the effect of the aberrant MET receptor activation leading to myogenic differentiation.

Taken together, constitutive activation of the MET receptor signaling blocks myogenic differentiation of RMS and enhances tumor proliferation, vascularization and metastasis (Figure 11A). Additionally we postulate that aberrant activation of MET signaling may be responsible for impairment of myogenic differentiation of MSC and as a consequence may lead to oncogenic transformation towards ERMS development (Figure 11B). In the literature it is postulated that RMS may derive from a differentiation defect of either MSC or myogenic progenitors [1]. We postulate that origin of RMS may be dependent on a subtype of the tumor. ARMS is usually associated with presence of PAX3/FKHR or PAX7/FKHR fusion genes [3], whereas ERMS does not display their expression. Based on our results and on the fact that PAX3 and PAX7 transcription factors are expressed in satellite stem cells [43], we suggest that ERMS may be derived from impaired differentiation of MSC. Nevertheless, in the future more detailed studies are required to convincingly verify our statement.

## MATERIALS AND METHODS

### Cell culture

RMS cell lines (SMS-CTR, RD, CW9019, RH30, RH28, RH18) have been kindly provided by Dr. PJ Houghton (Center for Childhood Cancer, Columbus, OH, USA) or have been ordered from American Type Culture Collection (ATCC, Manassas, VA, USA) and they were cultured in DMEM high glucose medium (PAA Laboratories GmbH, Pasching, Austria / Lonza Group Ltd., Basel, Switzerland) supplemented with 10% FBS (PAA / EURx, Gdansk, Poland), 50 μg/ml gentamicin (Lonza) at 37°C, 5% CO_2_ and 95% humidity. RMS cell lines were differentiated in DMEM low glucose medium (PAA / Lonza) supplemented with 2% horse serum (HS) (Gibco, BRL Grand Island, NY, USA) and 100 nM TPA (Sigma-Aldrich, St. Louis, MO, USA) for 7–10 days, as described previously [23]. For RMS cell culture in Matrigel the 12-well plate was covered with Matrigel (Corning Life Sciences – PZ HTL SA, Warsaw, Poland) and mixed with DMEM medium with 2% HS in proportion 1:1. Subsequently, 10 000 cells were seeded in Matrigel mixed with medium (with 2% HS) in proportion 1:4 and covered with medium with 2% HS. Cells were cultured for 7 days and medium was changed every 2–3 days. Morphology of the cells was assessed using microscope.

Adipose tissue derived mesenchymal stem cells (MSC) were isolated by us and characterized as described previously [58]. They were cultured in DMEM LG (PAA / Lonza) supplemented with 10% FBS for mesenchymal stem cells (STEMCELL Technologies Inc. Vancouver, BC, Canada), EGF (R&D Systems, Minneapolis, MN, USA), PDGF (Becton Dickinson, San Jose, CA, USA) and gentamicin.

Human myoblasts were isolated by us and characterized as described previously [59]. They were cultured in DMEM/F12 (Lonza) supplemented with dexamethasone, insulin (both from Sigma-Aldrich) 18% FBS, EGF (R&D Systems, Minneapolis, MN, USA), FGF (R&D) and HGF (R&D).

Human umbilical vein endothelial cells (HUVEC) have been ordered from Becton Dickinson Biosciences. They were cultured in M199 medium with HEPES (Invitrogen, Carlsbad, CA, USA) supplemented with 10% FBS (EURx), endothelial cell growth supplement (Sigma-Aldrich), heparin (Sigma-Aldrich) and gentamicin (Lonza).

### Production of viral vectors and transduction of cells

Plasmid encoding full length TPR-MET sequence was obtained from Addgene (Cambridge, MA, USA) and described previously [60]. Viral particles encoding GFP (GFP@pLenti6/UbC) and TPR-MET (TprMET@pLenti6/UbC) were produced using Vira Power Lentiviral Expression System (Invitrogen, Carlsbad, CA, USA), as described previously [61]. SMS-CTR cells and MSC were transduced with lentiviral vectors (at MOI = 10 and 20 respectively) in presence of 6 μg/ml polybrene (Sigma-Aldrich) and subsequently after 72 hours they were selected with 2.5 μg/ml blasticidin (InvivoGen, Toulouse, France) for two weeks. RH30 ARMS cells were transduced with lentiviral vectors encoding shMET and shLacZ, as described previously [22], [23].

### Transfection of cells with anti-miRs

SMS-CTR cells were transfected with 30 nM anti-miR miRNA Inhibitors (Ambion Inc., Austin, TX, USA) against miR-378a-5p and miR-378a-3p and negative control using Lipofectamine 2000 (Invitrogen) according to the manufacturer's instructions 72 h before the experiments. Subsequently RNA was isolated, as described below and cell culture media were collected from the cells cultured between 48 and 72 hours after the transfection.

### Treatment of the cells

SMS-CTR, RH30, RH28 and CW9019 cells were subjected to differentiation in DMEM (Lonza) with 2% horse serum (HS) (Gibco BRL) and with or without 100 nM TPA (Sigma-Aldrich) [23]. For evaluation of the phosphorylation level of AKT kinases, the SMS-CTR cells were seeded at density of 120 000 cells per well on 6-well plate. After 24 hours medium was changed for DMEM with 0.5% BSA. Next day, the cells were treated with 20 ng/ml HGF in fresh starving medium for 10 minutes at 37°C. Subsequently, protein was isolated, as described below.

### Flow cytometry

For evaluation of MET receptor level RMS cells were stained with monoclonal FITC-labeled anti-human HGFR/c-MET antibody, clone 95106 (R&D) or mouse IgG1 isotype control (R&D) and the results were analyzed using FACS Canto cytometer (Becton Dickinson) FACS Diva software (Becton Dickinson), as described previously [23].

### DNA and RNA isolation and reverse transcription

Genomic DNA was isolated using QIAmp DNA Blood Mini Kit (Qiagen, Hilden, Germany) according to vendor's protocol. The total RNA was extracted using Universal RNA purification Kit (EURx), according to vendor's protocol. For analysis of miRNA expression, total RNA was isolated with mirVana miRNA Isolation Kit (Ambion Inc., Austin, TX, USA). Total RNA from paraffin embedded tumor samples was isolated with RecoverAll™ Total Nucleic Acid Isolation Kit (Ambion). The reverse polymerase transcription of mRNA was performed using MMLV reverse transcriptase (Promega, Madison, WI, USA) according to the manufacturer's protocol. Reverse transcription of miRNA was performed with NCode VILO miRNA cDNA Synthesis Kit (Invitrogen), according to the vendor's protocol.

### PCR

Incorporation of TPR-MET transgene into genomic DNA was validated by PCR with a forward primer 5′-TGGACAATGATGGCAAGAAA-3′ and a reverse primer 5′-GAAGTGGATGGCTTTGGAAA-3′ using Taq PCR Master Mix (EURx), according to vendor's protocol. Expression of TPR-MET mRNA was evaluated by PCR with a forward primer 5′-GAGCCAATTTACAAGAACAAAGGA-3′ and reverse primer 5′-ATACTGCACTTGTCGGCATGAA-3′.

### Quantitative real-time PCR

Gene expression was determined by qRT-PCR analysis on ABI PRISM 7300 Sequence Detection System (Applied Biosystems, Foster City, CA, USA) using Blank qPCR Master Mix (EURx) and the following Taq-Man probes (Applied Biosystems): human: GAPDH (Hs99999905_m1), MET (Hs01565589_m1), VEGF (Hs00173626_m1), MMP9 (Hs00234579_m1), MYF5 (Hs00271574_m1), MYOD (Hs00159528_m1), MRF4 (Hs01547104_g1), MEF2A (Hs01050409_m1), MYOSTATIN (Hs00976237_m1), MYOGENIN (Hs01032275_m1), MYH2 (Hs00430042_m1), PAX3 (Hs00240950_m1), PAX7 (Hs00242962_m1), SNAI1 (Hs00195591_m1), RUNX2 (Hs00231692_m1), PPARG2 (Hs01115513_m1) and mouse: GAPDH (Mm99999915_g1).

For evaluation of miRNA expression by quantitative real-time PCR Sybr Green qPCR Master MIX (EURx) and universal reverse primer from NCode VILO miRNA cDNA Synthesis Kit (Invitrogen) were used with the following forward primers:

U6 snRNA: 5-CGCAAGGATGACACGCAAA TTC-3′

miR-1: 5′-GCTGGAATGTAAAGAAGTATGT ATAA-3′

miR-206: 5-TGGAATGTAAGGAAGTGTGTGG-3′

miR-133a-5p: 5-GCAGCTGGTAAAATGGAACCA AAT-3′

miR-133a-3p: 5′-TGGTCCCCTTCAACCAGCTG-3′

miR-133b: 5′-TTTGGTCCCCTTCAACCAGCTA-3′

miR-378a-5p: 5′-CCTGACTCCAGGTCCTGTGT-3′

miR-378a-3p: 5′-ACTGGACTTGGAGTCAG AAGG-3′

The mRNA expression level for all samples was normalized to the housekeeping gene GAPDH, whereas miRNA level was normalized to the housekeeping U6 snRNA level. Expression levels of miRNA and genes were quantified employing the 2 −ΔΔCt calculation or 2 −ΔCt using U6 snRNA or GAPDH a housekeeping controls.

### Analysis of protein (Western blotting and ELISA)

Protein was isolated with M-PER lysing buffer (Pierce, Rockford, IL, USA) and Western blot was done with anti-GAPDH rabbit mAb (Cell Signaling), anti-phospho-Akt (Ser473) rabbit mAb (Cell Signaling, Danvers, MA, USA), as described previously [22, 23]. ELISA for VEGF (R&D Systems) in media collected from the transfected cells was performed according to the vendor's protocol.

### Migration, chemotaxis and invasion through Matrigel

Confluent SMS-CTR cells were treated with DMEM medium with 0.5% BSA for 24 hours. Subsequently, a scratch was generated with a pipette tip. Starving medium was replaced every day. Photographs were analyzed using ImageJ software (National Institute of Health). Chemotaxis of cells to 20 ng/ml HGF (R&D System) and 100 ng/ml SDF-1 (Peprotech, Rocky Hill, NJ, USA) was evaluated using modified Boyden's chamber with 8 μm pore polycarbonate membrane inserts (Transwell; Corning Life Sciences – PZ HTL SA, Warsaw, Poland), as described previously [23]. 0.5% BSA DMEM medium was used as a negative control, whereas 10% FBS as a positive control. Similarly invasion of SMS-CTR cells through growth factor reduced Matrigel invasion inserts (Corning Life Sciences – PZ HTL SA, Warsaw, Poland) to 10% FBS, 20 ng/ml HGF, 100 ng/ml SDF-1, 0.5% BSA was also investigated, as described previously [23], using density of 5.0 × 10^4^ in 0.5 ml per one insert. Invasion of the cells treated with 20 ng/ml HGF was also investigated.

### Proliferation and mitochondrial activity

SMS-CTR cells were seeded on 24-well plates at density of 10 000 cells per well. After 24 hours medium was changed and 24, 48 and 72 hours later cells counted in a Burker hemocytometer chamber. To examine mitochondrial activity of the cells MTT test was done. 2000 of SMS-CTR cells were seeded on 96-well plates. After 24 hours medium was changed for either a medium with 10% FBS or 0.5% BSA and cells were incubated either in normoxia (21% O_2_ level) or at 5% O_2_ level for 24 hours. Afterwards mitochondrial activity of the cells was estimated with CellTiter 96^®^ AQueous One Solution assay (Promega), according to vendor's protocol.

### Angiogenic Matrigel assay *in vitro*

SMS-CTR cell lines were cultured on six- well-plates for 24 h in DMEM media with 2% FBS at 5% O_2_ level. Subsequently, the conditioned media were collected, and they were mixed with M199 medium with 2% FBS in a proportion of 1:1. For Matrigel assay, 50 μl of growth factor reduced Matrigel (BD Biosciences) was plated in a 96-well plate and incubated at 37°C for 30 min. HUVEC were detached, counted, and single-cell suspensions at a density of 10,000 cells per well in 200 μl of conditioned media and proper controls were plated on the Matrigel. Subsequently, endothelial tube formation was photographed 6 h after seeding. Formation of tubule-like structures was analyzed with Angiogenesis Analyzer for ImageJ (Carpentier G., Angiogenesis Analyzer for ImageJ (2012) available online: http://imagej.nih.gov/ij/macros/toolsets/Angiogenesis%20Analyzer.txt).

### Microscopy

Microscopic images were visualized with Oympus IX70 or Olympus BX51 microscopes (Olympus Corporation, Tokyo, Japan) and Canon EOS1100D digital photo camera (Canon Inc., Tokyo, Japan) or Olympus XC50 camera.

### *In vivo* experiments

5 × 10^6^ SMS-CTR and RH30 cells were injected subcutaneously into 6–8 weeks old NOD-SCID mice. Each experimental group comprised 4–5 animals and all the experiments were repeated two times. Tumor size was evaluated two times per week with a caliper and tumor volume was estimated with a formula V=D × d^2^ × 0.5 (V is the tumor volume, D is the biggest dimension; d is the smallest dimension). After 4 weeks the mice were killed and their tumors and bone marrow cells were harvested. After evaluation of tumor weight, they were fixed in formalin. Tumor sections were stained with hematoxylin-eosin to visualize tumor morphology and capillaries with erythrocytes inside them and after deparaffinization they were stained immunohistochemically, as described previously [23], with anti-Ki67 primary mouse monoclonal antibody to evaluate tumor proliferation (clon MIB-1; 1 : 75, DakoCytomation, Denmark, UK), anti-cleaved PARP (1:100, Abcam, Cambridge UK; ab32064) to visualize apoptosis and anti-CD31 antibody to visualize tumor vascularization (1:50, Abcam, ab28364). The sections were also stained immunofluorescently with anti-Ly6G/6C primary antibody to visualize murine neutrophils infiltrating tumor (clone RB6–8C5, 1:100, Biolegend, San Diego, California, USA) and subsequently with secondary goat anti-rat antibody conjugated with Alexa Fluor 555 (1:300, Life Technologies, Warsaw, Poland) and finally the slides were mounted in Vectashield Mounting Medium with DAPI (Vector Laboratories, Inc, Burlingame, CA, USA) The metastasis of SMS-CTR cells to the bone marrow was evaluated by real-time PCR using human GAPDH specific primers-probe set (Hs99999905_m1; Applied Biosystems) compared to murine GAPDH (Mm99999915_g1; Applied Biosystems). Expression of genes and miRNAs was evaluated in paraffin-embedded tumor samples, as described in previous section.

### Ethics statement

Human experiments were approved by the Local Bioethical Committee of the Collegium Medicum of the Jagiellonian University in Krakow, Poland (no. KBET/32/B/2014). Biopsies of rhabdomyosarcoma tumors were collected during routine surgery for preparation of paraffin-embedded samples and analysis of gene expression. Animal experiments were approved by I Local Ethics Committee in Krakow (no. 89/2009, 23/2013).

### Statistical analysis

Unless stated otherwise, results show mean +/− SEM of at least 2–4 independent experiments. Statistical analysis was performed by one-way ANOVA with Tukey post-test or student's *t*-test. Differences with a value of *p* < 0.05 were considered statistically significant. Correlations between genes in patients were calculated as Pearson's correlations.

### Supplementary information

Supplementary Information accompanies the paper on the website.

## SUPPLEMENTARY TABLE AND FIGURES


