# Supplementary material for: Constitutive activation of MET signaling impairs myogenic differentiation of rhabdomyosarcoma and promotes its development and progression
Source: Oncotarget. 2015 Sep 8;6(31):31378–98. doi: 10.18632/oncotarget.5145 (PMC4741613; doi:10.18632/oncotarget.5145)
Supplement: Supplementary file 1 [file oncotarget-06-31378-s001.pdf]

SUPPLEMENTARY TABLE AND FIGURES

Supplementary Table 1: Values of the Pearson correlation between MET and myogenic differentiation markers in RMS tumors.

	MET	MYF5	MyoD	MEF2A	myogenin	myostatin	MYH2
**MET**		**R = –0.68** *p* = 0.06	**R = 0.87** *p* = 0.005	**R = –0.33** *p* = 0.42	**R = –0.43** *p* = 0.29	**R = 0.26** *p* = 0.53	**R = -0.55** *p* = 0.16
**MYF5**	**R = –0.68** *p* = 0.06		**R = –0.58** *p* = 0.14	**R = –0.08** *p* = 0.86	**R = 0.16** *p* = 0.71	**R = 0.02** *p* = 0.96	**R = 0.45** *p* = 0.26
**MyoD**	**R = 0.87** *p* = 0.005	**R = –0.58** *p* = 0.14		**R = –0.37** *p* = 0.36	**R = –0.42** *p* = 0.30	**R = 0.27** *p* = 0.52	**R = –0.39** *p* = 0.34
**MEF2A**	**R = –0.33** *p* = 0.42	**R = –0.08** *p* = 0.86	**R = –0.37** *p* = 0.36		**R = 0.92** *p* = 0.001	**R = –0.49** *p* = 0.22	**R = 0.69** *p* = 0.06
**myogenin**	**R = –0.43** *p* = 0.29	**R = 0.16** *p* = 0.71	**R = –0.42** *p* = 0.30	**R = 0.92** *p* = 0.001		**R = –0.21** *p* = 0.62	**R = 0.89** *p* = 0.003
**myostatin**	**R = 0.26** *p* = 0.53	**R = 0.02** *p* = 0.96	**R = 0.27** *p* = 0.52	**R = –0.49** *p* = 0.22	**R = –0.21** *p* = 0.62		**R = 0.0005** *p* = 0.99
**MYH2**	**R = –0.55** *p* = 0.16	**R = 0.45** *p* = 0.26	**R = –0.39** *p* = 0.34	**R = 0.69** *p* = 0.06	**R = 0.89** *p* = 0.003	**R = 0.0005** *p* = 0.99	

Expression of genes was evaluated by qPCR in paraffin-embedded RMS specimens, $n = 8$, R = correlation coefficient, $p < 0.05$ considered as significant.

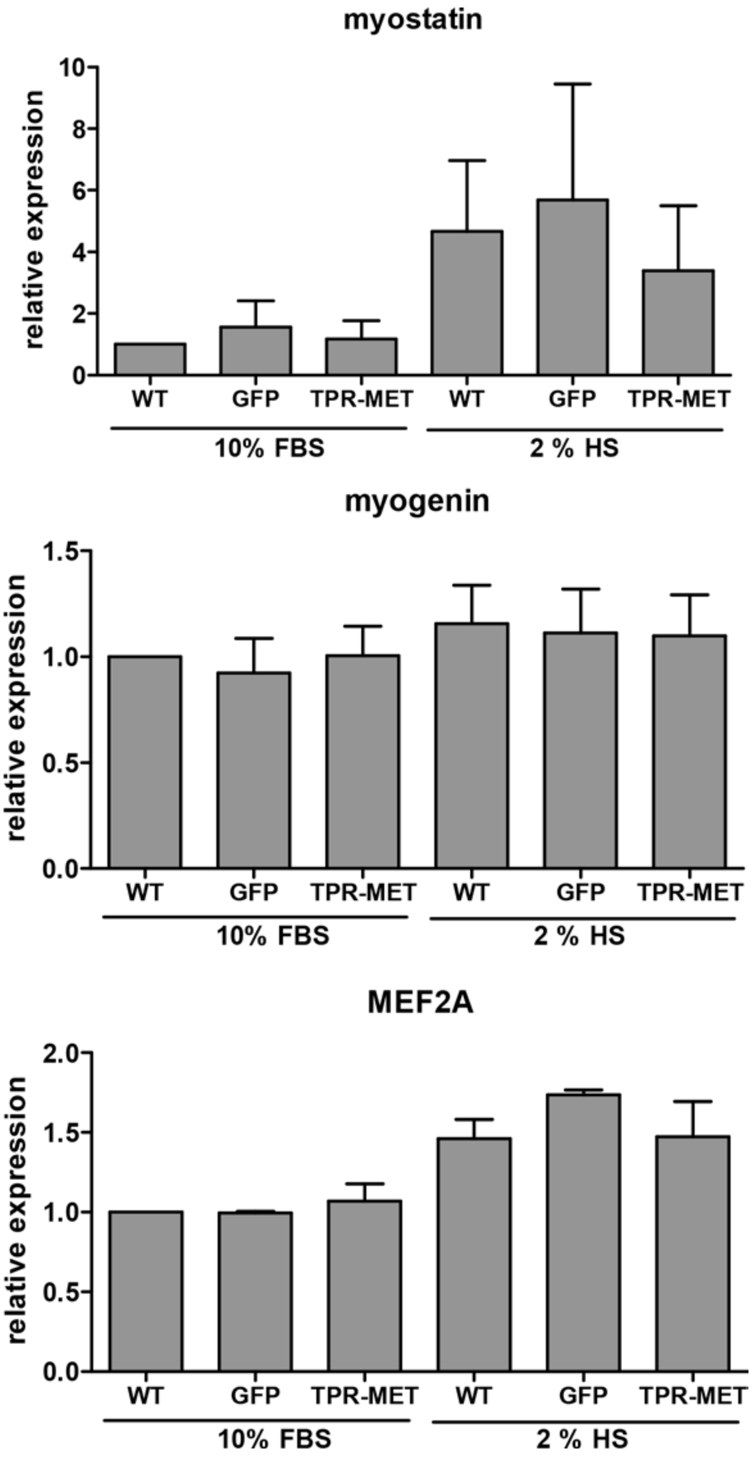

Supplementary Figure 1: Myostatin and MEF2A are slightly upregulated in SMS-CTR cells cultured in DMEM with 2% HS. Expression of myostatin, myogenin and MEF2A was evaluated by qPCR in samples from SMS-CTR cells, *n* = 2. Data in graphs are represented as mean +/− SEM.

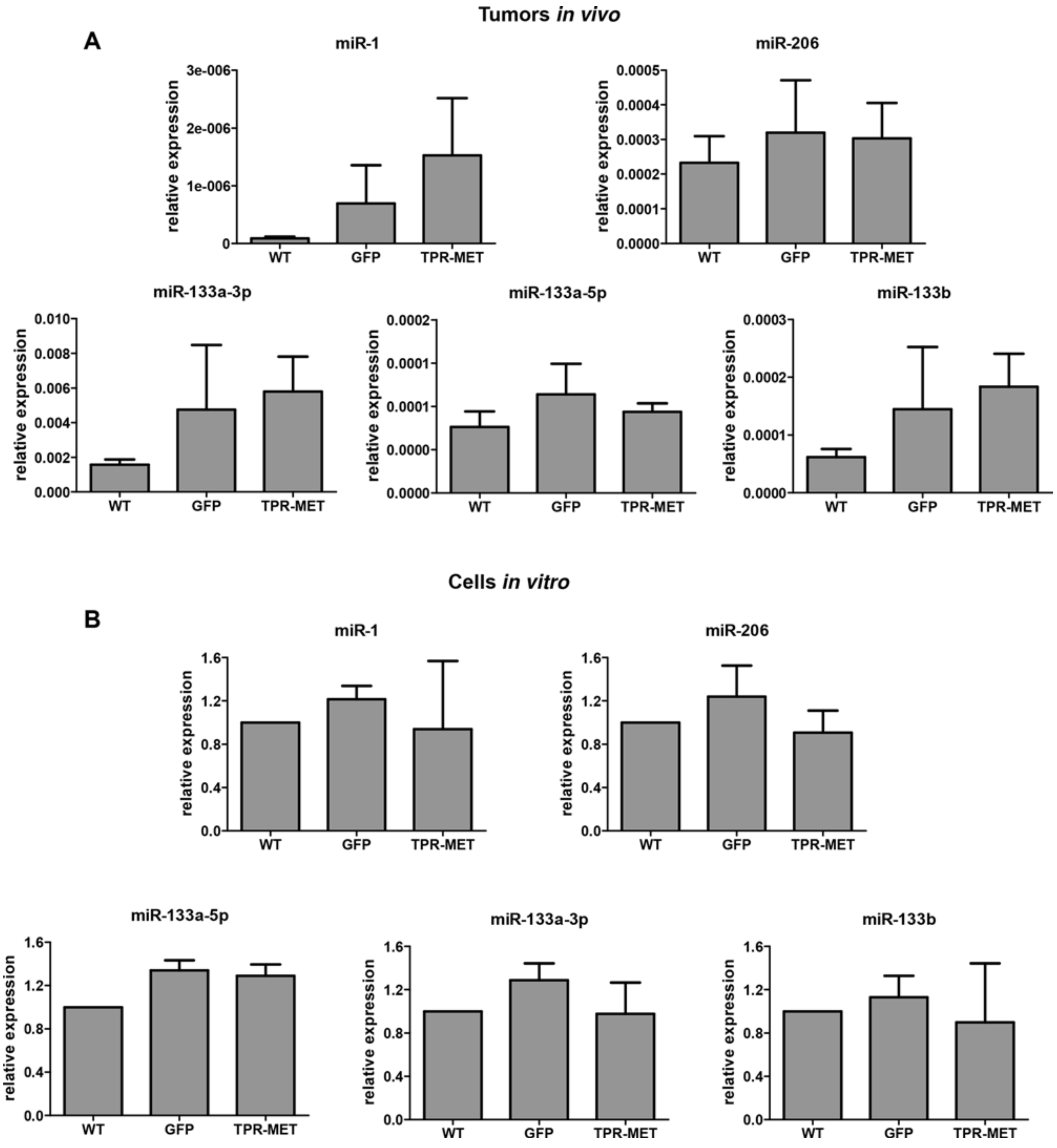

Supplementary Figure 2: The effects of TPR-MET are independent of myomiRs action. Expression of miR-1, miR-206, miR-133a-3p, miR-133a-5p and miR-133b was estimated by quantitative PCR in tumors formed by SMS-CTR cells after subcutaneous implantation to NOD-SCID mice, *n* = 4–5 **(A)** and in SMS-CTR cells *in vitro*, *n* = 2 **(B)** Data in graphs are represented as mean +/− SEM.

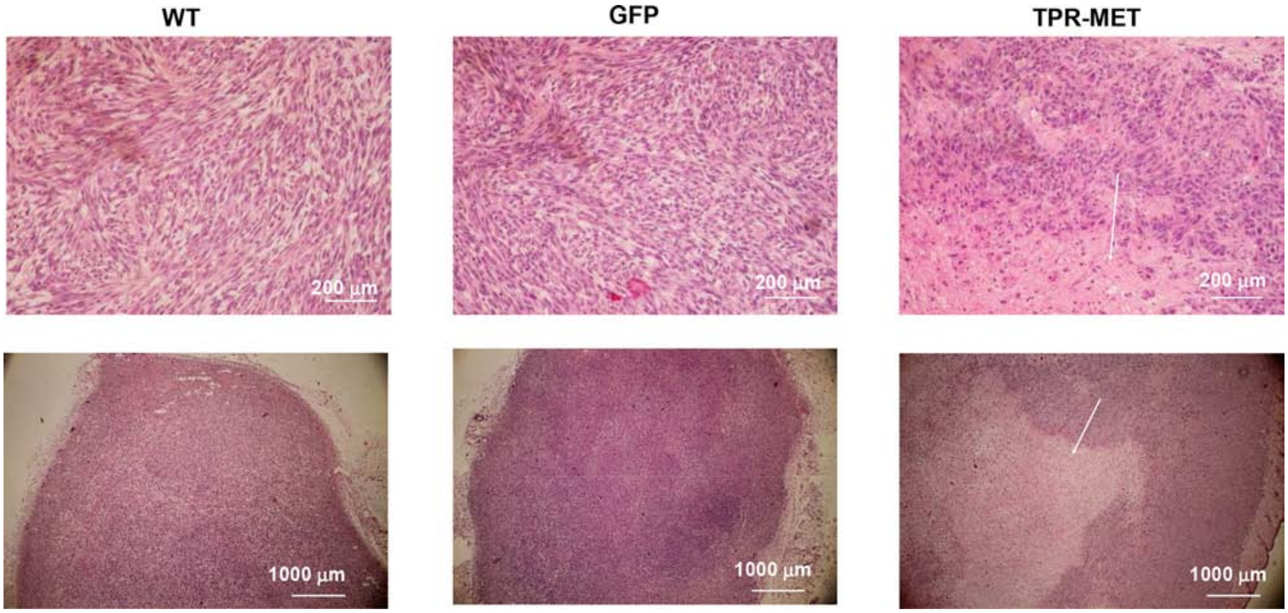

Supplementary Figure 3: Necrosis in tumors formed by SMS-CTR cells expressing TPR-MET. Representative images of tumors sections stained with hematoxylin-eosin show necrotic areas in TPR-MET tumors, which are indicated with arrows.
